# Instrumented Analysis of the Sit-to-Stand Movement for Geriatric Screening: A Systematic Review

**DOI:** 10.3390/bioengineering7040139

**Published:** 2020-11-06

**Authors:** Brajesh Shukla, Jennifer Bassement, Vivek Vijay, Sandeep Yadav, David Hewson

**Affiliations:** 1 System Science, Indian Institute of Technology Jodhpur, Jodhpur 342037, India; shukla.1@iitj.ac.in; 2 Human and Life Sciences, Laboratoire d’Automatique, de Mécanique et d’Informatique industrielles et Humaines, Université Polytechnique Hauts-de-France, 59300 Valenciennes, France; jennifer.bassement@uphf.fr; 3 Department of Mathematics, Indian Institute of Technology Jodhpur, Jodhpur 342037, India; vivek@iitj.ac.in; 4 Department of Electrical Engineering, Indian Institute of Technology Jodhpur, Jodhpur 342037, India; sy@iitj.ac.in; 5 Institute for Health Research, University of Bedfordshire, Luton LU1 3JU, UK

**Keywords:** biomedical monitoring, functional screening, Kinect, RGB camera, IMU, sit-to-stand

## Abstract

The Sit-to-Stand (STS) is a widely used test of physical function to screen older people at risk of falls and frailty and is also one of the most important components of standard screening for sarcopenia. There have been many recent studies in which instrumented versions of the STS (iSTS) have been developed to provide additional parameters that could improve the accuracy of the STS test. This systematic review aimed to identify whether an iSTS is a viable alternative to a standard STS to identify older people at risk of falling, frailty, and sarcopenia. A total of 856 articles were found using the search strategy developed, with 12 articles retained in the review after screening based on the Preferred Reporting Items for Systematic Reviews and Meta-Analyses (PRISMA) guidelines. Six studies evaluated the iSTS in fallers, five studies in frailty and only one study in both fallers and frailty. The results showed that power and velocity parameters extracted from an iSTS have the potential to improve the accuracy of screening when compared to a standard STS. Future work should focus on standardizing the segmentation of the STS into phases to enable comparison between studies and to develop devices integrated into the chair used for the test to improve usability.

## 1. Introduction

The age of the world’s population is increasing in all countries due to advances in medical care, with developed countries such as Japan already having 26% of its population aged over 65 years, while the United Kingdom currently has 18% of its population aged over 65 years [1]. The problem of an ageing population is not restricted to developed countries, but also of developing countries; for example, 13% of India’s population is predicted to consist of older people by 2050 [1]. Given the size of the Indian population, this equates to over 220 million older people. Although medical progress has increased the expected lifespan, many older people, although living longer, are still suffering from many age-related conditions that decrease their healthy lifespan. Functional decline has been reported as the most common phenotypical expression of diseases that are present in older people [2]. The incidence of the two most common conditions related to functional decline, frailty and sarcopenia, are becoming increasingly prevalent, with 18% of the Indian population reported to have sarcopenia [3]. The prevalence of sarcopenia has been reported to be roughly twice that of frailty [4], with prevalence increasing with age, with over 50% of octogenarians reported to have sarcopenia [5].

A side effect of frailty and sarcopenia conditions is an increased risk of falls, with around one third of adults aged over 65 years falling each year [6]. This figure rises to over 50% in the very old, for those aged over 80 years [7]. It seems likely, therefore, that the general trend towards an ageing population will result in an increase in the number of fallers. In one recent study, people with sarcopenia had a 50% increase in all-cause mortality over a 15-year period [8].

Although falls are a problem for older people, studies have shown that, provided people at risk of falling can be detected, a falls prevention program can successfully decrease the rate of falling [9]. Likewise, if older people in the early stages of the frailty phenotype or with pre-sarcopenia can be identified, a physical activity-based intervention could stop or even reverse the onset of these conditions [10].

One way of screening for such conditions is to use functional geriatric screening tests, such as the Timed-Up-and-Go (TUG) or the Sit-to-Stand Test (STS). The STS movement is a commonly used screening test due to its ability to assess the strength and power of the lower limbs, and can therefore be used to predict falls [11,12], while it is also one of the preferred tests of physical function in sarcopenia [13]. The STS is becoming widely used due to the simplicity of use, because it requires only a chair and a stopwatch, with results of the test recorded as either the time taken to perform five consecutive chair stands (5STS) or the number of chair stands able to be performed in 30 s (30STS).

Rather than limit the information collected from functional screening tests to a simple measure of the time taken to perform the task, a number of investigators have begun to add technological devices to tests in order to provide more precise information about the physical capacity of the person being tested. For instance, an instrumented version of the TUG is now widely used, with the qTUG recommended for use by the National Institute for Health and Care Excellence in the U.K. [14]. Several studies have been published in which the standard STS has been augmented with the use of technology such as cameras or body-worn sensors [15]. There has been one previous review of instrumented STS, however this was performed in 2014, with many newer studies having been published [16]. In addition, this review focused only on motion sensor devices, with no studies of video technology included in the review, while the effectiveness of the iSTS were not evaluated with respect to any diagnostic accuracy. Therefore, the aim of this systematic review, was to identify whether an instrumented version of the STS offers a better alternative to a standard test to identify older people at risk of falling, frailty, and sarcopenia.

## 2. Materials and Methods

The search for articles was carried out based on the Preferred Reporting Items for Systematic Reviews and Meta-Analyses (PRISMA) [17]. The electronic databases searched were MEDLINE, CINAHL, Web of Science, and IEEE Xplore. Hand searches of reference lists of the selected articles were used to identify other relevant studies. Publication date was limited to dates between 1994, when the STS was first published by Guralnik and colleagues [18] as part of the Short Physical Performance Battery (SPPB), to August 2020.

Searches were limited to the title and abstract of articles, with the following key words used:

1. Older people: Old* OR elder* OR geriatric* OR senior* adj5 (people OR adult* OR person*)
2. Sit-to-Stand: Sit-to-stand OR stand-to-sit OR chair stand OR STS OR 5STS OR 30STS OR CST
3. Technology: sensor* OR instrument* OR accelerometer* OR gyroscope* OR magnetom* OR ICT OR device OR smartphone OR motion capture OR video OR Kinect OR camera

### 2.1. Types of Studies

All types of quantitative study designs were included in the review.

### 2.2. Types of Participants

Studies were limited to community-dwelling people aged 60 years and older. Any studies in which participants with a specific disease or condition, other than frailty, sarcopenia, or being fallers, such as Parkinson’s Disease or dementia, were excluded. Articles in which instrumented STS were developed, but not evaluated on older people were not included.

### 2.3. Primary Outcomes

The primary outcome of the studies selected needed to include an evaluation of iSTS performance to discriminate between older people with and without one of the three chosen health factors or conditions; fall risk, frailty, or sarcopenia.

### 2.4. Inclusion and Exclusion Criteria

Studies with subjects aged under 60 years or with a specific medical condition were excluded. The STS test performed needed to be a recognized variation of the STS, such as the 5STS or 30STS, with studies in which only the sit–stand transition was reported being excluded.

### 2.5. Data Extraction

Articles were identified by the combined keyword searches for each database separately, with duplicates removed. Two reviewers (BS and DH) screened the titles and abstracts of these articles to identify relevant studies to retain for full-text screening. Any articles for which a full-text version could not be obtained was excluded from the review. Data were extracted from the full-text versions of the studies separately by both reviewers, with information subsequently pooled. Data extracted included study design and characteristics, the variant of the STS, the technology used, and the ability of the instrumented device to discriminate between participants with frailty, sarcopenia, or fallers from non-fallers. 

Cohen’s d was used as a measure of magnitude for the differences between groups [19]. This comparison was used for groups such as fallers and non-fallers, or different frailty levels, and is calculated as:

(1) $$\mathrm{Cohen}’s\;d\;=\frac{\mu_{2}-\mu_{1}}{pooled\;\left(\sigma \right)}$$

where $\mu_{1}$ and $\mu_{2}$ are means of fallers and non-fallers, respectively. The estimate of pooled (standard deviation) is given by:

(2) $$pooled\;\left(\sigma \right)=\sqrt{\frac{\left(n_{1}-1\right)\sigma_{1}^{2}+\left(n_{2}-1\right)\sigma_{2}^{2}}{n_{1}+n_{2}-2}}$$

where $\sigma_{1}$ and $\sigma_{2}$ are standard deviations of fallers and non-fallers, respectively, and $n_{1},\;n_{2}$ denote the respective sample sizes. The p-value was obtained from the t-distribution table as the Cohen’s d-statistic followed a t-distribution. When Cohen’s d is used with small sample sizes there is an upwards bias that can be corrected using the Hedges’ *g* estimation, which produces an unbiased estimate of the population effect size [20], as given by:

(3) $$g=d\left[1-\frac{3}{4N-9}\right]$$

Correlations were used to indicate the strength of association between tests of physical function and parameters extracted from the iSTS. Owing to the skewed distribution of correlation coefficients, Fisher’s *Z* transformation was used [21] before combining correlations, as given by:

(4) $$Z=0.5\;ln\left[\frac{1-r}{1+r}\right]$$

The inverse *Z* transformation was then used to convert Fisher’s *Z* back into a correlation coefficient, as given by:

(5) $$r=\left[\frac{\left(e^{2z}-1\right)}{\left(e^{2z}+1\right)}\right]$$

One of the assumptions for a classification algorithm is the uniform distribution of classes, whereby both the classes should have the same number of instances. A problem often occurs when there are imbalances between classes, such as having more non-fallers than fallers. The general measure of accuracy typically used can misrepresent the results due to the influence of the majority class. A better variable to use is the Area Under the Curve (AUC) of the receiver operating characteristic (ROC) curve, which plots True Positive Rate (TPR) against False Positive Rate (FPR). The AUC provides an aggregate measure of performances across all possible classification thresholds and can also be interpreted as the probability that the model ranks a random positive example more highly than a random negative example. The standard error of AUC values can be calculated using the Hanley–McNeil approach [22].

The inverse variance weighting method was used to produce pooled estimates of effects [23]. Summary of finding tables were used to report the magnitude of effects for each outcome measure, while the Grading of Recommendations Assessment, Development, and Evaluation (GRADE) system was used to rate the quality of evidence from the included studies [24].

### 2.6. Quality Appraisal

The criteria adopted to appraise articles for this review was the method of Loney and colleagues [25] and Sanderson and colleagues [26], and modified by Payette and colleagues [27], which is suitable for observational studies. This method uses ten criteria, with each one scored as zero or one, and the total score taken as an index of methodological quality. The questions used for this appraisal were: (1) Are the recruitment sources described? (2) Are the criteria for exclusion or inclusion well defined? (3) Are required sample size calculations presented? (4) Is the method of calculating the iSTS parameters clearly described? (5) Is the evaluation procedure clearly described? (6) Has the outcome measure for the health condition been adequately described? (7) Does the results section present a minimum of descriptive information about the participants, such as age (mean, range, or standard deviation) and gender? (8) Are the statistical analyses for evaluation of the association between iSTS parameters and the healthcare condition described? (9) Are effect sizes reported with measures of precision? (10) Are the study’s limitations adequately presented? Studies that scored at least 5 out of 10 were considered to be satisfactory and were included in the review [25].

## 3. Results

### 3.1. Article Selection

**Figure 1:**
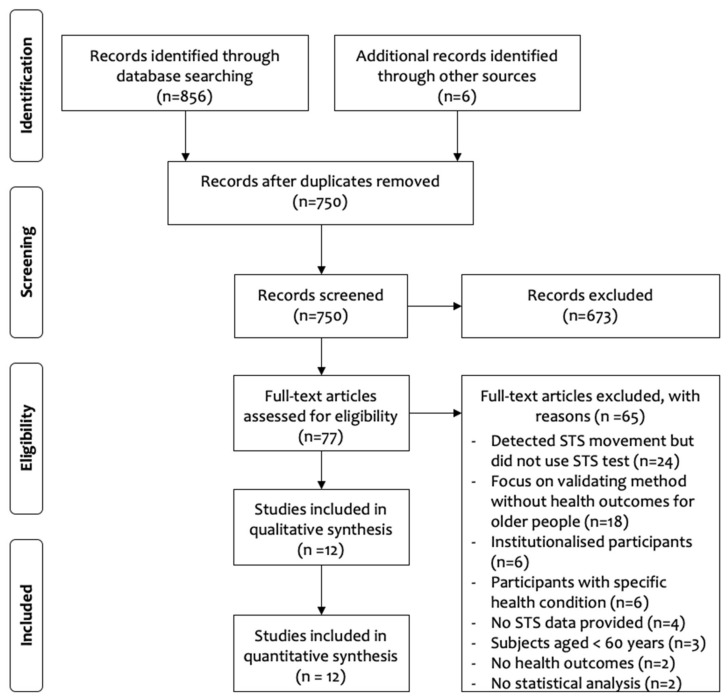
Preferred Reporting Items for Systematic Review and Meta-Analyses (PRISMA) flow chart of study selection [17].

A PRISMA flowchart of the search is shown in Figure 1. A total of 856 articles were retrieved from the databases searched, with a further six articles identified from other sources. After duplicates were removed, 750 articles remained for title and abstract screening, resulting in 673 articles being removed for not meeting the inclusion criteria. The remaining 77 articles underwent full-text appraisal, with 65 articles rejected. The remaining 12 articles were retained for the systematic review, with characteristics of the articles presented in Table 1.

### 3.2. Study Characteristics

The 12 studies selected used seven different approaches to obtain an instrumented STS. Nine studies used sensors that were attached to the body. Four studies from two research groups used Inertial Measurement Units (IMUs), with three of the studies from the same research group using a single IMU placed on the third lumbar vertebra [28,29,30]. The remaining study used five IMUs, three of which were placed on the lower limbs, with the remaining two placed on the fifth lumbar vertebra and the sternum [31]. Two studies from the same research group used two triaxial accelerometers placed on the thigh and sternum [32,33], while one group used a hybrid device consisting of a triaxial accelerometer and a pressure sensor worn in a pendant around the neck [34]. The remaining study used accelerometery, utilising a smartphone worn on a waist belt [35]. The study in which a sensor was attached to the body used a linear position transducer that was attached to the belt by a cable [36]. The remaining three studies used a Microsoft Kinect sensor placed perpendicular to the chair [37], four force plates integrated into the chair [38], and a chair equipped with load cells and a light detection and ranging (LiDAR) sensor [39].

In total, 1701 participants were included in the 12 studies, none of which evaluated people with sarcopenia. Seven studies compared fallers and non-fallers, one of which used fallers with hip fractures, while one study used both fall and frailty classification in the same participant group [31]. All studies in which falls were used to classify participants used retrospective falls history. Both the duration and the number of falls used in the definitions of falls varied between studies, with the definitions used for fallers shown in Table 2. Six studies studied frailty with three different screening tools used. In four studies, a modified version of the Fried frailty phenotype [40] was used to compare frail, pre-frail and robust participants. The remaining two studies used the Groningen Frailty Indicator [41] and the FRAIL scale (Fatigue, Resistance, Ambulation, Illnesses, & Loss of Weight) [42], with the definitions used for frailty shown in Table 2.

With respect to the STS test, three different versions were used in the 12 selected studies. Eight studies focused on the 5STS test, three studies used the 30STS, while one study used a less-common version of the STS in which only three repetitions were performed [38]. All studies in which the 30STS was used contained participants with frailty, rather than falls as the health condition of interest.

### 3.3. Evaluation of Fallers and Non-Fallers

The results for fallers and non-fallers for the seven studies in which fallers and non-fallers were compared are summarized in Table 3. The typical difference between fallers and non-fallers corresponded to a small effect using Cohen’s d [19]. The time to complete different phases of the STS was compared by three studies, with no differences observed for stand-to-sit, while a small effect was observed for sit-to-stand time [32].

A range of different parameters were calculated for the iSTS, with these parameters classified as force/power, frequency, and velocity. The parameters for force/power and velocity during the STS showed moderate differences between groups, while those for frequency showed small differences. 

Several studies reported correlations as a measure of the association between STS parameters and functional capacity tests for strength, balance, and mobility. Similarly, with the differences between groups, the largest correlations were found for force/power and velocity variables, with moderate effects typically reported. Finally, three studies reported models with respect to discriminating or classifying between fallers and non-fallers [31,33,36]. Two machine learning algorithms, a support vector machine (SVM) and logistic regression (LR), were used for classification with a range of different parameters used in the models reported, meaning pooled estimates could not be produced. With respect to classification accuracy, the results were modest.

### 3.4. Evaluation of Frailty Sub-Groups

In one study, the robust participants were not aged 60 years and above, therefore the results for this study have not been included for the frailty comparisons [29]. Two of the studies used participants from the same study with different parameters for the iSTS, but the same results for the number of cycles performed in the STS [28,30]. Accordingly, the results of only one of these studies for this parameter were included. There was a significant difference between frail and pre-frail, with frail participants performing fewer STS cycles (Frail 6.24 ± 2.53, Pre-frail 8.16 ± 2.42, d = 0.79; *p* < 0.05) [28]. There was also a significantly greater number of STS actions performed by robust compared to pre-frail participants (Pre-frail 8.16 ± 2.42, Robust 9.86 ± 3.00, d = 0.63; *p* < 0.05) [28]. Both of these effects can be considered as moderate.

Comparisons between frail, pre-frail, and robust participants are summarized in Table 4. With respect to frail vs. pre-frail groups, large effects were observed for velocity and phase time, with moderate effects for force/power and acceleration. Comparing pre-frail and robust groups, moderate effects were found for force/power and phase time, with small effects for acceleration and velocity. Two studies examined the association between frailty scores and the iSTS, with one reporting a non-significant correlation of −0.16 for the association between chair rise peak power and the Groningen Frailty Index [34]. In the other study, the STS score from the SPPB was used, where higher scores indicated better performance [18]. This study reported a significant negative correlation of −0.41 with the frailty score, where higher scores indicated higher levels of frailty [39]. Finally, two studies classified participants into frailty categories using STS parameters [28,31], with classification accuracy varying from 52.9% to 89.9%.

## 4. Discussion

### 4.1. Overview

The aim of this systematic review was to determine whether an instrumented version of the STS offers a better alternative than a standard STS with respect to the identification of older people at risk of falling, frailty and sarcopenia. A total of 12 articles were identified, all of adequate quality, with six evaluations of fallers and seven evaluations of frailty. The methods used to classify participants in the studies as fallers and non-fallers varied widely, making it difficult to aggregate the findings. There were also differences between the studies in which frailty was used as the health outcome measure, although most studies did use a variant of the Fried frailty phenotype.

None of the articles selected used an iSTS to compare differences between older people with and without sarcopenia. In one respect, this could be expected given that the STS is one component of the SPPB, which is one of the tests of physical function used in sarcopenia screening such as the updated version of the European working group on sarcopenia in older people (EWGSOP) sarcopenia algorithm [43]. On the other hand, given that the STS is already used for sarcopenia screening, the use of iSTS parameters related to power and velocity might offer an alternative to some of the other tests already used, thus decreasing the time needed for screening.

The quality of evidence for all the studies included in this review can be considered low using the GRADE system recommended by the Cochrane Library [24]. It should be noted that the GRADE system classifies all observational studies as low evidence, which suggests that further studies are needed.

### 4.2. iSTS and Fallers

The iSTS evaluations produced parameters of different types, of which the most common were temporal, power and force, velocity, and acceleration. With respect to fallers, the results of temporal parameters tended to be similar to those of the time taken for the entire STS. This could indicate that, rather than there being a particular phase of the STS such as the sit-to-stand transition that is difficult for fallers to perform, it might be equally difficult to perform all phases. However, another reason for the lack of differences observed could be that many different methods of dividing the STS into phases were used, while some studies did not segment the STS at all [34,36]. The most phases identified in the STS was four [37], which included the sit-to-stand transition, stand-to-sit transition, and standing and sitting phases. The smallest number of phases used was two, although one of these methods only used the sit-to-stand and stand-to-sit transitions, discarding the time spent in between [31,32,33], while the other method contained only the preparation for the sit-to-stand transition and the actual transition, without analysing the stand-to sit [38]. It seems clear that, in order to compare studies more readily, it would be worthwhile standardizing the phases used to make it easier to compare results between studies.

One potential benefit of using an iSTS is the possibility of identifying differences related to force, power, velocity, and acceleration, which cannot be obtained from the STS. This was shown by the results of this review in which consistently useful results were obtained for power and velocity parameters, with several moderate differences observed. This was particularly the case for the sit-to-stand transition, when power needs to be generated to ensure the person can stand up. Indeed, previous work has already shown that the lower-limb power produced can be predicted using a logistic regression equation that contained STS performance body mass [44]. Other work has shown that an equation using STS time, leg length and body mass can predict knee extensor strength and even the cross-sectional area of the quadriceps [45]. Given that these models predict muscle power based only on the time taken to perform an STS test or the time taken to complete a number of cycles, it would be interesting to investigate whether the power directly calculated from an iSTS would give better results than the predictive equations of these studies.

With respect to the technologies used to instrument the STS in fallers, the majority of studies used body-worn sensors that were either IMUs or triaxial accelerometers. Other methods used included force plates, a linear transducer, and several studies by the same research group using a Kinect sensor. When the different techniques were compared, the best results in terms of differences between fallers and non-fallers were found for the chair equipped with force plates [38], although it should be noted that these results were for fallers with hip fractures, so further work is needed for this technology with different subject groups. Classification of fallers and non-fallers was only reported by two studies, one of which did not include accuracy, making it difficult to compare. Although the accuracy from an IMU-based system was only moderate [31], an excellent odds ratio (OR) was obtained with the linear transducer [36] to distinguish fallers from non-fallers.

### 4.3. iSTS and Frailty

The majority of the frailty results were from the same research group, with the other two studies only providing comparisons between frailty categories and a single correlation between the iSTS and the overall frailty score. This means that the results for frailty were almost exclusively from the one group using the Kinect sensor and the 30STS [28,29,30], rather than the 5STS, which was used by the studies on fallers. In the case of the 30STS, rather than the time taken to perform a test, the number of STS cycles performed in 30 s is used to define STS. Frailty comparisons were then made between the three categories of frail, pre-frail and robust, with moderate effect sizes observed for the number of STS cycles between frail and pre-frail and between pre-frail and robust groups. However, when the time spent in the three phases of the STS used in this analysis were considered, larger differences were observed between frail and pre-frail categories than between pre-frail and robust. The other iSTS parameters had similar results, with a greater number of large effects observed between frail and pre-frail than between pre-frail and robust.

When the iSTS was used in two studies to classify people into frailty categories, the best classification was obtained in a study using the Kinect sensor, which had 90% accuracy when using a decision tree model with iSTS parameters, which was much better than the 53% classification for the number of STS cycles alone. A model in which multiple parameters are used is more likely to perform well than a single-factor model, but nevertheless, the result demonstrated the utility of the iSTS approach. However, the results of the classifications used should be interpreted with caution, due to issues such as imbalance within the groups used in the studies. For instance, in one of the two studies in which frailty was classified, only 31 of the 718 participants (4%) were frail [28]. Imbalanced datasets can be dealt with using appropriate methods such as the Synthetic Minority Oversampling TEchnique (SMOTE) [46]. However, the frailty classification study included in this review did not report the use of any model validation techniques, unlike the study in which fallers were classified using a 10-fold cross-validation technique [47]. Previous studies on other devices used for older people, such as sensor-based fall risk testing, have also reported that the accuracy of models has been inflated due to methodological issues in feature selection and validation [48].

In terms of sensor locations, there are some advantages on sensors that are not body-worn in terms of acceptability and convenience. For instance, the requirement to place the sensors on the body means that issues of acceptability and ease of placement need to be considered, as well as the type of clothes worn by the person being evaluated. There has been little research into user preferences in this area, although in one report, the preferred location for wearable sensors was reported to be the wrist [49], which could be less effective at detecting whole body movement during the STS cycles [50]. It is also possible that results would differ if sensors were incorrectly placed on the body, which would be particularly relevant for the comparison of results between individuals and across sessions. In contrast, sensors that do not need to be worn, such as the Kinect, might be quicker and easier for testing, including standardization.

In addition to the articles chosen for this systematic review, there were many articles rejected at full-text screening as they did not include a specific version of the STS, and instead used a standalone STS movement, meaning that the iSTS and STS could not be compared. Despite this, it would be worth examining the parameters developed in such studies to see whether some of the methods used could be incorporated into 5STS or 30STS tests for clinical use. 

There were also many articles that were not selected because they were focused on validating new methodologies. Although some of these articles were earlier work by authors whose articles were included in this review, many were from other research teams that could potentially provide valuable information on the STS when tested in populations of older people with falls history, frailty, and sarcopenia. Finally, one area that was not evaluated in any of the studies was change between the different STS cycles within an STS test. For instance, in the 30STS, it might be worth comparing parameters from the first STS cycles with those from later cycles to determine whether any fatigue effect is present.

## 5. Conclusions

The results of this systematic review have identified the emergence of the instrumented STS as an area with great potential to improve the detection of strength-related conditions such as physical frailty, and to assess the risk of falling in older people. A range of parameters extracted from an iSTS, including velocity and power, are better at differentiating between groups than the time taken for the STS alone. However, there is a need for more standardization in terms of the parameters extracted from the iSTS, as well as the way in which segmentation of the STS into phases is performed. This would ensure that results of studies can be directly compared to produce variables that could be used for geriatric screening. In terms of sensor location, issues of sensor acceptability should be investigated, while the development of more devices that are non-invasive, such as those integrated into the chair or the environment, could be beneficial to decrease any problems of standardization of protocols and sensor placement for body-worn devices.

## Figures and Tables

**Table 1:** Characteristics of the articles accepted after full-text screening.

Authors	Technology	Version	Subjects ^1^	Age (Years)	Condition	Quality
Coni et al. (2019)	Triaxial accelerometers	5STS	304 (53.6%)	80.9 ± 6.4	Fallers	6
Doheny et al. (2011)	Triaxial accelerometers	5STS	40 (60%)	71.4 ± 7.3	Fallers	6
Doheny et al. (2013)	Triaxial accelerometers	5STS	39 (59%)	Fallers: 74.9 ± 7.0	Fallers	7
				Non-fallers: 68.4 ± 6.2		
Ejupi et al. (2016)	Kinect sensor	5STS	94 (70.2%)	79.7 ± 6.4	Fallers	9
Greene et al. (2014)	IMU	5STS	124 (73.4%)	Non-frail: 73.7 ± 6.0	Frailty, Fallers	8
				Frail: 77.8 ± 6.4		
				Fallers: 76.0 ± 6.2		
				Non-fallers: 75.8 ± 6.8		
Houck et al. (2011)	Force plates in a chair	3STS	28 (71.4%)	Control: 69.4 ± 10.9	Fallers	9
				Hip fracture: 76.4 ± 7.1		
Jung et al. (2019)	Load cells in a chair and laser	5STS	40 (62.5%)	74.3 ± 5.4	Frailty	9
Millor et al. (2013)	IMU	30STS	47 (44.7%)	Frail: 85 ± 5	Frailty	6
				Prefrail: 78 ± 3		
				Robust: 54 ± 6		
Millor et al. (2014)	IMU	30STS	431 (N/S)	Frail: 79 ± 6	Frailty	5
				Pre-frail: 73 ± 5		
				Robust: 74 ± 5		
Millor et al. (2017)	IMU	30STS	431 (N/S)	Frail: 79 ± 6	Frailty	6
				Pre-frail: 73 ± 5		
				Robust: 74 ± 5		
Vincenzo et al. (2018)	Linear position transducer	5STS	98 (62.2%)	77.5 ± 7.3	Fallers	9
Zhang et al. (2017)	Triaxial accelerometer, pressure sensor	5STS	25 (80.0%)	79.7 ± 5.7	Frailty	7

^1^ Percentage of female subjects; N/S: Not Stated.

**Table 2:** Definitions used for fallers and frailty.

Authors	Definition Used for Falling and Frailty
Coni et al. (2019)	Faller: ≥2 falls in previous 12 months
Doheny et al. (2011)	Faller: ≥2 falls in previous 5 years, or previous fall requiring medical attention, or fear of falling, or cardiovascular risk factor for falls
Doheny et al. (2013)	Faller: ≥2 falls in previous 12 months or fall requiring medical attention
Ejupi et al. (2016)	Faller: fall in previous 12 months
Greene et al. (2014)	Faller: ≥2 falls in previous 12 months or fall requiring medical attention Frailty: Fried phenotype used to classify participant as robust, pre-frail, or frail
Houck et al. (2011)	Faller: hip fracture from fall within previous 12 months, no longer receiving physical therapy
Jung et al. (2019)	Frailty: score on the Korean version of the FRAIL scale
Millor et al. (2013)	Frailty: Fried phenotype used to classify participant as robust, pre-frail, or frail
Millor et al. (2014)	Frailty: Fried phenotype used to classify participant as robust, pre-frail, or frail
Millor et al. (2017)	Frailty: Fried phenotype used to classify participant as robust, pre-frail, or frail
Vincenzo et al. (2018)	Faller: fall in previous 12 months
Zhang et al. (2017)	Frailty: Groningen Frailty Indicator

**Table 3:** Summary of findings for fallers vs. non-fallers.

Population: Anyone Classified as A Faller					
Settings: Clinical or Laboratory					
Evaluation: Instrumented Sit-to-Stand Test					
Comparison: Non-Fallers					
Outcomes	Illustrative Example		Effect Size (95% CI)	Number of Participants (Studies)	Evidence Quality (GRADE)
	Fallers	Non-Fallers			
iSTS total time	16.8 s	14.3 s [37]	0.42 ^1^ (0.10, 0.74)	172 (3 studies)	Low
iSTS stand-to-sit time	0.45 s	0.45 s [32]	0.03 ^1^ (−0.33, 0.39)	172 (3 studies)	Low
iSTS sit-to-stand time	0.49 s	0.41 s [32]	0.38 ^1^ (0.10, 0.66)	172 (3 studies)	Low
iSTS force/power	6.2 W/Kg	7.3 W/kg [36]	0.56 ^1^ (0.36, 0.76)	126 (2 studies)	Low
iSTS frequency	13.1 Hz	11.3 Hz [32]	0.45 ^1^ (0.16, 0.73)	39 (1 study)	Low
iSTS velocity	0.41 m/s	0.50 m/s [36]	0.56 ^1^ (0.35, 0.77)	192 (2 studies)	Low
Physical function vs. iSTS force/power	r = 0.499 [38] peak GRF ^3^ vs. gait speed		0.46 ^2^ (0.35, 0.51)	27 (1 study)	Low
Physical function vs. iSTS velocity	r = 0.533 [37] STS velocity vs. knee extension		0.43 ^2^ (0.33, 0.53)	94 (1 study)	Low
Physical function vs. iSTS time	r = 0.316 [37] STS time vs. knee extension		0.31 ^2^ (0.21, 0.42)	94 (1 study)	Low
Classification using iSTS parameters	72.6% accuracy in classifying fallers [31]		Not estimable	261 (3 studies)	Low

^1^ Pooled estimate using Cohen’s *d*; ^2^ Pooled estimate using Fisher’s Z-transformed correlation coefficients; ^3^ Ground Reaction Force.

**Table 4:** Summary of findings for comparisons by frailty level.

Population: Anyone Classified as Frail						
Settings: Clinical or Laboratory						
Evaluation: Instrumented Sit-to-Stand Test						
Comparison: Frail vs. Pre-Frail and Pre-Frail vs. Robust						
Outcomes	Illustrative Example			Effect Size (95% CI)	Number of Participants (Studies)	Evidence Quality (GRADE)
	Frail	Pre-Frail	Robust			
STS cycles	6.24	8.16	9.86 [28]	0.79 ^2^ (0.40, 1.17)	431 (2 studies)	Low
				0.63 ^3^ (0.43, 0.83)		
iSTS phase time ^1^	1.67 s	1.30 s	1.10 s [30]	0.81 ^2^ (0.61, 1.00)	237 (2 studies)	Low
				0.53 ^3^ (0.43, 0.63)		
iSTS force/power	38.1 W	65.4 W	88.7 W [28]	0.73 ^2^ (0.51, 0.95)	237 (2 studies)	Low
				0.72 ^3^ (0.56, 0.88)		
iSTS acceleration	1.01 m/s^2^	1.28 m/s^2^	1.36 m/s^2^ [28]	0.72 ^2^ (0.56, 0.88)	237 (2 studies)	Low
				0.44 ^3^ (0.28, 0.59)		
iSTS velocity	0.48 m/s	0.61 m/s	0.68 m/s [30]	0.83 ^2^ (0.64, 1.02)	237 (2 studies)	Low
				0.46 ^3^ (0.27, 0.65)		
Classification of groups using iSTS parameters	AUC = 0.934 for classification of frail participants using decision tree model [28]			0.83 ^4^ (0.82, 0.85)	361 (2 studies)	Low

^1^ Example shown for stand-to-sit time; ^2^ Pooled estimate using Cohen’s d for comparison between frail and pre-frail groups; ^3^ Pooled estimate using Cohen’s d for comparison between pre-frail and robust groups; ^4^ Pooled estimate of classification accuracy using AUC.
